# Cross-sectional survey of compliance behaviour, knowledge and attitudes among cases and close contacts during COVID-19 pandemic

**DOI:** 10.1016/j.puhip.2023.100370

**Published:** 2023-02-15

**Authors:** Patricia M. Kearney, Danko Stamenic, Katarzyna Gajewska, Margaret B. O'Sullivan, Sarah Doyle, Orlaith O'Reilly, Claire M. Buckley

**Affiliations:** a School of Public Health, University College Cork, Ireland; b Department of Public Health, Health Services Executive, HSE South, Ireland; c Clinical Design and Innovation, Health Services Executive, Ireland

## Abstract

**Objectives:**

A key public health intervention is self-isolation for cases and restriction of movement for contacts. This study aimed to identify predictors of compliance behaviour and describe knowledge and attitudes among cases and contacts identified by the national Contact Management Programme to inform the global public health response.

**Study design:**

Secondary data analysis of anonymised cross-sectional survey data on national sample of cases and close contacts.

**Methods:**

A sample of 1000 cases and 1000 contacts was calculated to estimate compliance within a margin of error of 3% with 95% confidence. A telephone survey administered by trained interviewers collected information on socio-demographics, compliance behaviours, knowledge, and attitudes to COVID-19 from cases and close contacts. Data analysis included chi-squared statistics and multivariable logistic regression.

**Results:**

Most cases and contacts complied with public health guidance with similar characteristics in those who did and did not comply. Reasons for non-compliance included exercise, medical appointment, shopping, and work. Cases and contacts reported high levels of understanding about symptoms of COVID-19 and satisfaction with available information.

**Conclusion:**

Achieving high compliance with public health guidance is feasible and requires political leadership, policy changes and practical solutions.

## 1 Introduction

SARS-CoV-2, caused significant morbidity and mortality worldwide and unparalleled economic and social disruption [1,2]. The initial global response depended on non-pharmaceutical interventions (NPIs) to protect population health. Governments varied considerably with correspondingly differing outcomes [3]. The European Centre for Disease Prevention and Control (ECDC) guidelines categorise NPIs as individual, environmental, or population level measures [4]. Measures implemented included movement restrictions, partial closure or closure of schools and businesses, quarantine in specific geographic areas and international travel restrictions [5,6]. In a study comparing restrictions phased restrictions, informed by local epidemiological indicators and supported by a robust contact tracing and testing system, were key components of the COVID-19 pandemic response [7].

The initial public health response to the COVID-19 pandemic in Ireland focused on a strategy of containment [8]. In March 2020, a national Contact Management Programme (CMP) was established to notify results to people tested (or their nominated person), to identify and manage contacts of people with COVID-19, and to provide public health guidance [9]. During the first surge, the Irish government moved to a mitigation approach. Following the initial emergency measures, the government developed a road map for reopening society and businesses [10]. Subsequently, the government issued a new plan for ‘*living with COVID-19’* moving from a short-term emergency response to a medium-term approach [11].

Public health interventions to control disease transmission have varying levels of evidence of effectiveness [12]. The World Health Organization (WHO) identifies contact tracing, the process of identifying, assessing, and managing people exposed to a disease to prevent onward transmission, as an essential public health tool for controlling infectious disease outbreaks [13]. The use of quarantine to control infectious diseases goes back centuries [14]. Today, many countries have the legal authority to impose quarantine which, in accordance with Article 3 of the International Health Regulations (2005), must be fully respectful of the dignity, human rights and fundamental freedoms of persons [15]. While contact tracing and quarantine are considered critical activities to reduce transmission [2,16], the effectiveness of contact tracing depends on many factors including compliance [17,18]. A rapid evidence review conducted early in the COVID 19 pandemic identified fourteen studies which assessed compliance to quarantine during earlier infectious disease outbreaks including mumps, Severe Acute Respiratory Syndrome (SARS) and Ebola. The review identified a range of factors influencing compliance including sociodemographic characteristics, risk perception, understanding of disease risk, and practical considerations concluding that compliance is difficult, variable and depends on the specific setting or population [20]. Over the course of the pandemic, countries and regions worldwide implemented contact tracing systems with varying levels of compliance. A recent review identified varying political, social, economic, and cultural factors which affected the initial response in different global regions [21]. Given these factors, it is perhaps unsurprising that countries and regions reported widely varying levels of compliance during the COVID-19 pandemic identifying factors including age, gender and understanding of disease transmission as key factors influencing compliance [[22], [23], [24], [25], [26], [27], [28], [29]]. As Ireland implemented a national contact management programme at the start of the pandemic, an opportunity exists to assess compliance behaviour in different sociodemographic groups and to explore the impact of knowledge and attitudes. While the Garda Síochána (the police service) in Ireland was given new powers to enforce public health restrictions including population level travel restrictions, individual’s compliance with self-isolation and restriction of movements was based on a voluntary approach.

This paper aims to describe compliance behaviour, knowledge, and attitudes in Ireland among cases and close contacts to further understanding of factors contributing to compliance thereby informing future pandemic preparedness.

## 2 Methods

### 2.1 Survey instrument

The survey is reported using the Checklist for Reporting of Survey Studies (CROSS) [30]. The survey questions (supplementary material) were developed iteratively based on national standards as per Health Protection Surveillance Centre (HPSC) guidance, international evidence, and best practice. Individual survey items were derived initially from a set of questions developed by the COVID-19 Rapid Survey of Adherence to Interventions and Responses (CORSAIR) study to measure behaviours, attitudes, beliefs, and consequences pertinent to the COVID-19 pandemic [31,32], and the International Assessment of COVID-19-related Attitudes, Concerns, Responses and Impacts in Relation to Public Health Policies (iCARE) study [33]. The interviewers were provided with dedicated training including importance of obtaining full and complete answers and emphasising to cases/contacts the importance of providing accurate information.

### 2.2 Study design

This is a secondary data analysis of anonymised cross-sectional survey data collected by trained social interviewers by telephone. The sampling frame was the national CMP, which manages approximately 97% of cases reported in Ireland weekly.

### 2.3 Sampling technique

The sample was identified in a single-stage. Individual level data (mobile phone number, first name, age group, gender, and county of location) for cases and contacts were extracted from the CovidCare Tracker (CCT) IT system for dates at least 10 days (cases) or 14 days (contacts) since date of contact by the CMP [9]. The county information was collapsed into five regions based on the four provinces with Dublin separate from the rest of Leinster. Deceased cases, and cases and contacts resident in congregate settings were excluded. The cases and contacts files were provided to Ipsos MRBI, a market research company. Following removal of incorrectly formatted phone numbers (e.g., missing digit), files were split into batches of 50 and assigned to interviewers. During November 9th-16th 2020 for cases, and November 11th-17th 2020 for close contacts, the numbers were called by interviewers. Numbers were called at least three times before being considered uncontactable.

### 2.4 Measurements

At the time of the survey, national guidance was for individuals with symptoms to self-isolate and arrange a test. Individuals who tested positive for COVID-19, ‘cases’, were advised to self-isolate for 10 days from the date of onset of symptoms (or date of test if asymptomatic). Their ‘close contacts’, people exposed to an infectious case of COVID-19, were advised to restrict their movements for 14 days from the last date of exposure and attend for testing on day 5 and day 10. The term ‘restriction of movement’ is used as that is the terminology used in Ireland [34]. Self-isolation was defined as ‘staying in your room’ whereas restriction of movement was defined as ‘staying at home’. Compliance was assessed based on self-report of adherence with self-isolation for cases and with restriction of movements for contacts. Symptomatic cases were asked to report their compliance behaviour before the test, while waiting the test result, and after the test. No distinction was made based on the type of home or household. Symptoms were based on self-report of symptoms of COVID-19.

Exposure Definitions: A case was defined as being notified by the CMP of positive result in the defined period. A close contact was defined based on identification by a case as a close contact during their infectious period including (i) anyone that the case had face to face contact for longer than 15 min in any setting (less than 2 m or six and half feet contact); (ii) household contacts defined as living or sleeping in the same house, individuals in shared accommodation that share kitchen and bathroom facilities as well as sexual partners and (iii) anyone with whom the case shared a closed space with for longer than 2 h in any setting (e.g.workplace, school, social venue, transport).

Covariates: Age and gender were extracted from the CCT IT system. Age (continuous variable) was recategorized into 10-year age bands. The survey included questions on employment status, education level, English language native speaker and fluency, number of people in the household, presence of children in the household, personal history of chronic illness knowledge of public health guidance, satisfaction with information available, personal risk perception and worry about COVID-19.

#### 2.4.1 Outcomes

Self-isolation after being informed of test result: For what reasons, if any, they left home after being contacted.

Self-isolation after developing symptoms: For what reasons, if any, they left home after they developed symptoms.

Requesting a test: cases were asked about reasons for seeking test.

Sharing details of close contacts: Whether they shared details of close contacts. For those who reported not sharing, reasons were sought.

Restriction of movement after being alerted to close contact status: For what reasons, if any, they left home after being contacted.

Requesting a test: contacts were asked whether a test had been requested and number of tests.

### 2.5 Statistical analysis

A target sample size of 1000 cases and 1000 contacts was selected to provide an estimate of the true prevalence of compliance to within a margin of error of 3% with 95% confidence. Descriptive statistics including numbers and percentages for categorical variables and means and standard deviations (SD) (or medians and IQR depending on the distribution) for continuous variables describe the characteristics overall and stratified by compliance status. Student’s t-test was used for comparison of means while Pearson’s chi-squared test and Fisher’s exact test were used for comparison of counts between different groups of participants. Binomial logistic regressions were used to investigate factors associated with non-compliance with self-isolation (cases) and movement restriction (contacts). Non-compliance for cases was defined as non-compliance before the test, before the result or after the result. Univariate regression assessed the association between individual characteristics and non-compliance. Multivariable regressions included adjustment for non-native English language speaker, employment status (working vs not working), presence of dependent child in the household, gender, age, highest educational or professional qualification (degree or higher vs less than degree), having a chronic illness, and living alone. Statistical analyses were conducted in R version 4.1.1 (R Core Team, 2021) [35].

### 2.6 Ethical considerations and data protection

The processing of personal health information by the Medical Officer of Health and/or their team and sharing of the information with the responsible person is permissible in order to protect individuals and the public under the Health Acts 1947 and 1953; Infectious Diseases Regulations 1981, and subsequent amendments to these regulations [36,37]. The survey was undertaken in response to a request from the National Public Health Emergency Team. A Data Privacy Impact Assessment for the survey was prepared in consultation with the HSE Data Protection Officer. The unconsented data was shared with Ipsos MRBI using a Data Confidentiality Agreement. Verbal informed consent was obtained by the interviewers at the start of the interviews. For those aged 16–18 years, parental consent was sought to conduct the interview directly with the minor. For younger children, the survey was conducted with the parent or guardian. All personal identifiers were removed before data transfer to University College Cork (UCC) servers using a secure file transfer service. Ethical approval for this secondary data analysis of anonymised survey data was provided by the Social Research Ethics Committee in UCC.

## 3 Results

**Fig. 1:**
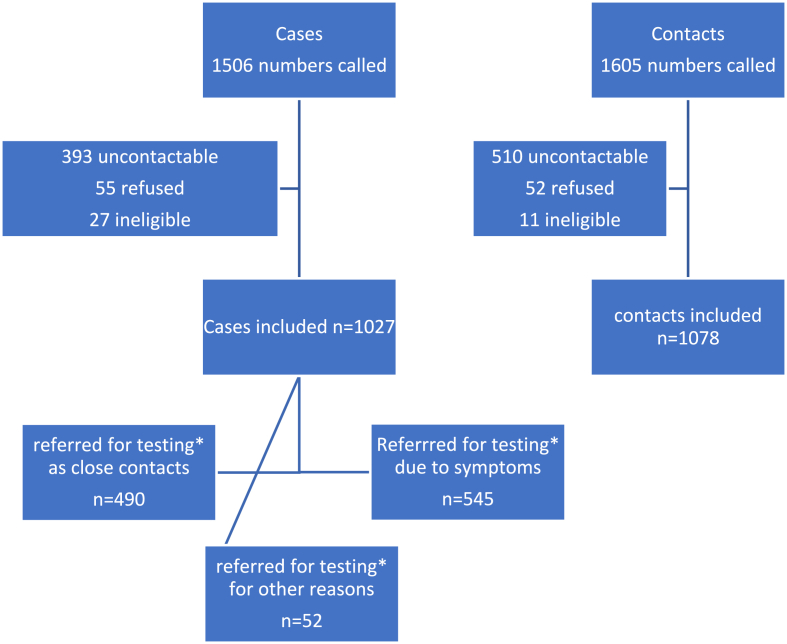
Inclusion of cases and contacts *60 cases reported being a close contact and having symptoms.

Over three thousand phone numbers were called to achieve the target sample of one thousand cases and one thousand contacts (Fig. 1). Among the 1027 cases, most reported their indication for testing as presence of COVID-19 symptoms (n = 545) or close contact with a case (n = 490), including 60 people reporting both symptoms and close contact. Other indications included international travel (n = 2), workplace serial testing (n = 27), feeling generally unwell (n = 7), worry (n = 31) and testing prior to hospitalisation for a procedure or operation (n = 8). The characteristics of cases and contacts are summarised in Table 1 with similar characteristics among those who did and did not comply with the public health guidance (**Webtable 1).**

**Table 1:** Characteristics of cases and contacts.

		Cases N = 1027	Close Contacts N = 1078
Gender	*Male*	553 (53.8)	501 (46.6)
	*Female*	474 (46.2)	575 (53.4)
Age	*Mean (SD)*	36.1 (17.4)	36.3 (19.22)
Occupation	*Employed*	607 (59.1)	611 (56.7)
	*Unemployed*	74 (7.2)	72 (6.7)
	*Student/pupil*	215 (20.9)	216 [20]
	*Retired*	59 (5.7)	81 (7.5)
	*Home/family*	55 (5.3)	66 (6.1)
	*Other*	17 (1.7)	32 (3.0)
Highest education level	*Primary*	39 (3.8)	75 (7.0)
	*Secondary*	451 (44.3)	473 (44.4)
	*Tertiary*	528 (51.9)	517 (48.5)
Household size	*Median, IQR*	4 [3,5]	4 [3,5]
Child in household	*Child present*	424 (43.7)	444 (43.6)
	*None*	546 (56.3)	574 (56.4)
Long-term health condition	*Present*	203 (19.8)	203 (18.9)
	*None*	822 (80.2)	870 (81.1)
Native English speaker	*Yes*	970 (94.4)	1032 (95.7)
	*No*	57 (5.6)	46 (4.3)

### 3.1 Compliance

High levels of compliance with self-isolation were reported by symptomatic individuals before attending for a test across a range of characteristics **(**Fig. 2 (a)). Among symptomatic cases, those who self-isolated prior to the test reported an average of 2.41 days between onset of symptoms and their test compared with 3.98 days among those who did not self-isolate while awaiting a test (p < 0.001). While most age groups had >80% compliance, it was 66.7% in those aged 55–64 years and 75.6% in those >65 years. Overall, 979 (95.3%) cases complied with self-isolation after the test while waiting for the test result and 992 (96.6%) complied with self-isolation once informed of their test result. High levels of compliance (>90%) were observed among cases before and after the test result across different sociodemographic characteristics including age, gender, region, occupation, and education level (Fig. 2 (b) and 2 (c)). Among the 1078 contacts, 86.5% complied with restriction of movements with similar levels across age, gender, and region (Fig. 2 (d)).

**Fig. 2 (a):**
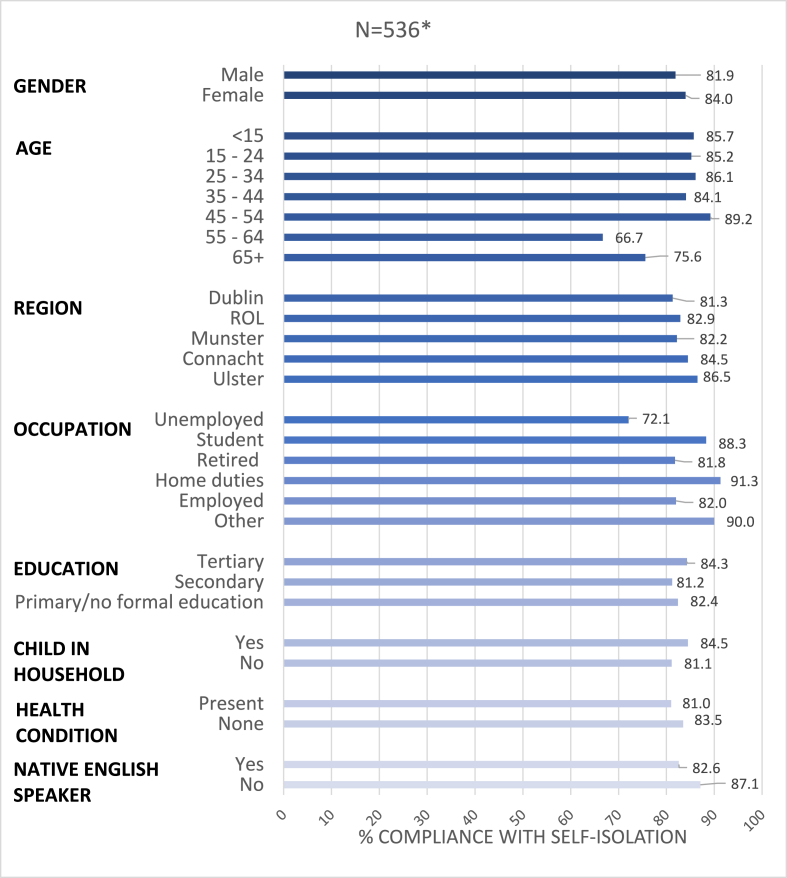
**Symptomatic cases compliance with self-isolation before the test** *Nine symptomatic cases did not provide an answer to the question **“**After you started feeling this way, but BEFORE you were tested, did you leave your home for any reason?” ROL = rest of Leinster.

**Fig. 2 (b):**
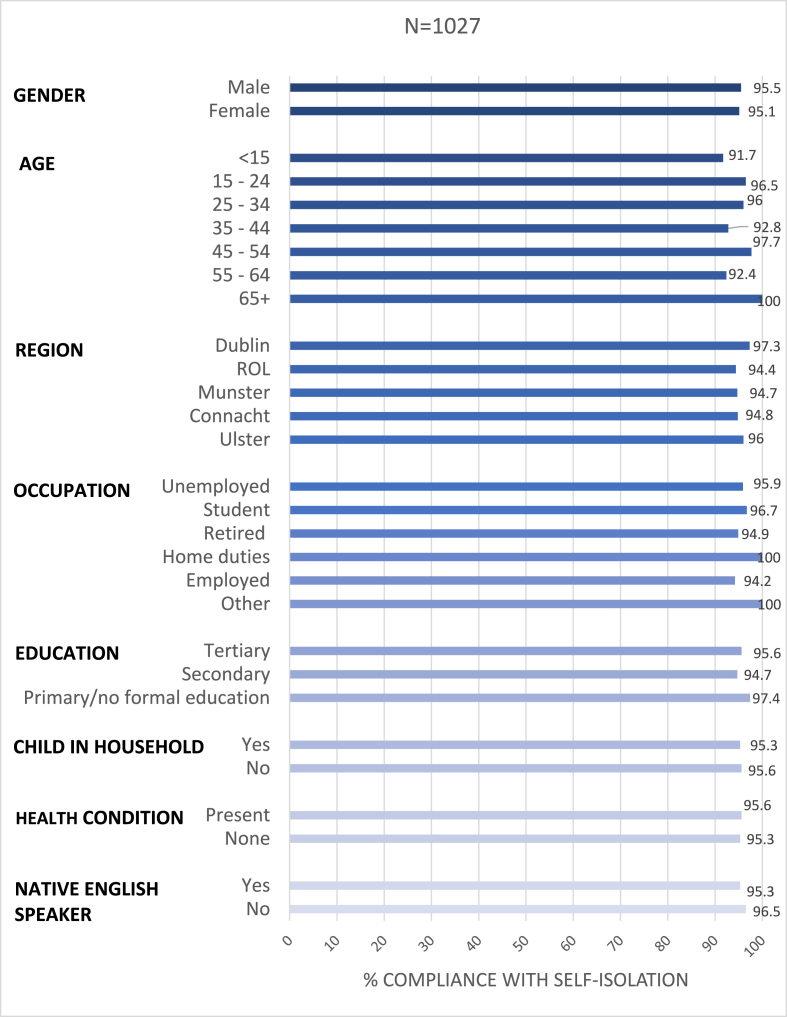
**Compliance among cases after testing and before the test result**.

**Fig. 2 (c):**
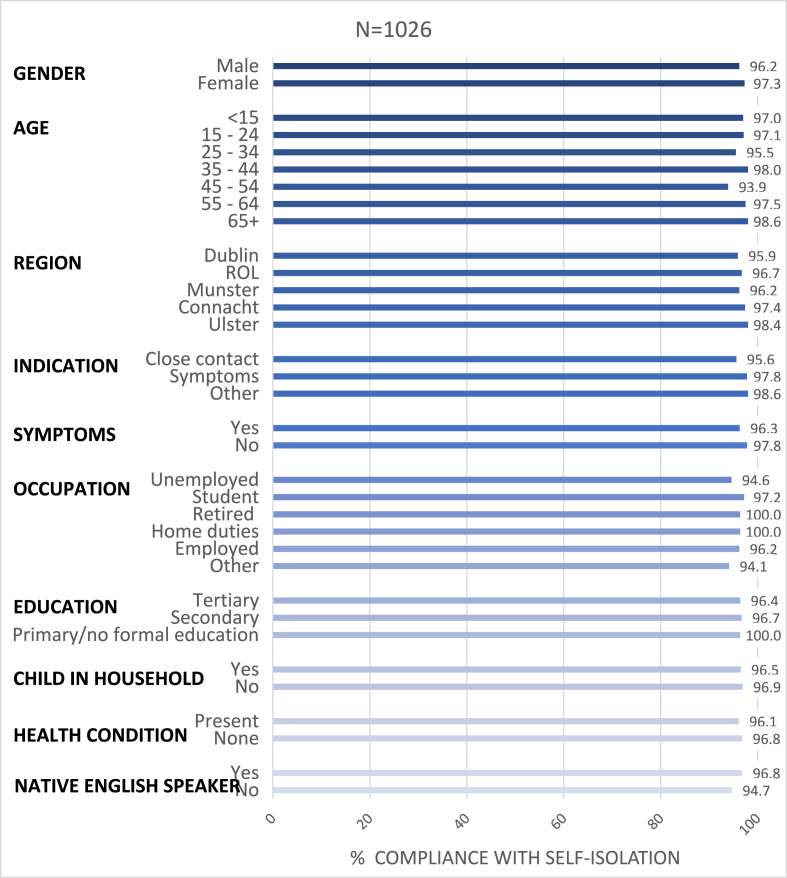
**Compliance among cases after testing positive**.

**Fig. 2 (d):**
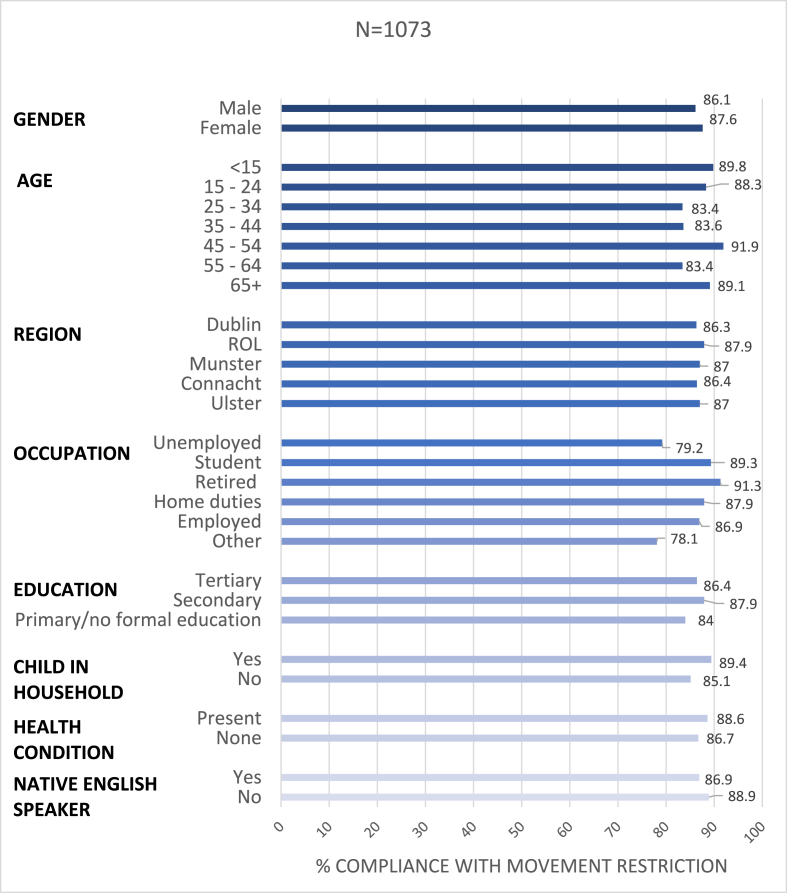
**Compliance with restriction of movements by contacts**.

Most (97%) cases reported providing details of all close contacts. Of the 26 cases who reported some or no contacts, reasons included not remembering all contacts (n = 13, 50%), being unsure what counted as a close contact (n = 3, 11.5%), embarrassment (n = 1, 3.8%) and worry about consequences for others (n = 3, 11.5%). Of the 1074 contacts, 561 had a single (day zero) test and 501 had two tests (day zero and day 7) with 12 reporting not having a test.

### 3.2 Non-compliance

In the multivariable logistic regression model, there was no evidence of an association between any of the sociodemographic characteristics and non-compliance with exception of age which had a u-shaped relationship **(Webtable 2)**.

Reasons reported by cases and contacts for non-compliance with self-isolation or restriction of movements are summarised in Table 2. Considering all cases, <1% attended work and 1.2% went to a shop before the test. After receiving their detected result, <1% of cases reported attending work, <1% reported attending a medical appointment, <1% reported socialising and <2% reported going for exercise or a walk. Overall, among close contacts, 3% reported attending work, 5.5% reported attending shops and 5.6% reported going for a walk/exercise with other reasons representing ≤1%.

**Table 2:** Reasons for Leaving House reported by cases and contacts at different time points in testing pathway among those who did not comply with public health guidance.

Reason for leaving home^a^	Cases			Contacts
	Before Test	Before Result	After Result	Before Test
	N = 91	N = 48	N = 34	N = 140
	N (%)	N (%)	N (%)	(%)
Shopping for groceries or medicines	21 (22.8)	13 (27.1)	0 (0)	40 (28.6)
Shopping other than groceries or medicines	4 (4.3)	0 (0)	0 (0)	19 (13.6)
Work or job related	42 (45.7)	8 (16.7)	2 (5.9)	32 (22.9)
Medical appointment	10 (10.9)	34 (70.8)	6 (17.6)	11 (7.9)
Exercise or a walk	12 (13.2)	11 (22.9)	19 (55.9)	60 (42.9)
Social event or to visit family or friends	7 (7.6)	23 (6.3)	1 (2.9)	5 (3.6)
Bring children to school	2 (2.2)	27 (56.3)	0 (0)	4 (2.9)
School/college	3 (3.3)	25 (52.1)	0 (0)	8 (5.7)
Other	2 (2.2)	3 (3.3)	6 (17.6)	5 (3.6)

^symptomatic cases only.

a More than one reason could apply.

### 3.3 Knowledge and attitudes

Cases and contacts demonstrated a lack of understanding of some public health guidance with only one-half of cases and contacts correctly reporting need for symptomatic people to self-isolate while waiting a test. However, over three-quarters of cases and nearly nine-tenths of contacts recognised need to self-isolate once tested positive (Table 3). There was no evidence of a difference in knowledge levels among those who did or did comply. Contacts largely agreed or strongly agreed with the statement that they had enough information about symptoms, testing, and self-isolation and that they received enough information from the contact tracer. Cases and contacts reported moderate levels of perception of risk of COVID-19 to themselves with higher levels of risk perception for family and friends and people in Ireland.

**Table 3:** Knowledge and attitudes among cases and contacts about COVID-19.

		Cases				Contacts			
		Overall	Self-isolated N = 992	Did not isolate	p-value	Overall	Restricted Movement	Did not Restrict	p-value
		N = 1027	N (%)	N = 34		N = 1078	N = 933	N = 140	
		N (%)		N (%)		N (%)	N (%)	N (%)	
When having symptoms of COVID-19, but before being tested, one should:	*Limit contacts outside home*	51 [5]	49 (4.98)	2 (6.1)	0.65	101 (9.4)	85 (9.2)	16 (11.7)	0.49
	*Stay at home*	421 (41.4)	410 (41.7)	11 (33.3)		451 (42.2)	388 (41.8)	60 (43.8)	
	*Self-isolate in a room*	545 (53.6)	524 (53.3)	20 (60.6)		517 (48.4)	455 (49)	61 (44.5)	
When tested for COVID-19 and waiting for test result, one should:	*Limit contacts outside home*	63 (6.1)	60 (6.05)	3 (8.8)	0.64	68 (6.3)	54 (5.8)	14 (10.1)	0.13
	*Stay at home*	306 (29.8)	297 (30.0)	9 (26.5)		446 (41.6)	384 (41.3)	59 (42.4)	
	*Self-isolate in a room*	657 (64)	634 (64.0)	22 (64.7)		558 (52.1)	491 (52.9)	66 (47.5)	
When tested positive for COVID-19, one should:	*Limit contacts outside home*	69 (6.7)	66 (6.65)	3 (8.8)	0.37	7 (0.7)	6 (0.6)	1 (0.7)	0.77
	*Stay at home*	172 (16.7)	164 (16.5)	8 (23.5)		106 (9.9)	90 (9.7)	15 (10.8)	
	*Self-isolate in a room*	786 (76.5)	762 (76.8)	23 (67.6)		960 (89.5)	834 (89.7)	123 (88.5)	

	Mean (SD)							
Someone could spread coronavirus to other people, even if they do not have symptoms yet^a^	4.8 (0.59)	4.8 (0.6)	4.82 (0.5)	0.85	4.74 (0.7)	4.7 (0.73)	4.83, (0.5)	0.03
Perceived risk of COVID-19 to self	3.06 (1.39)	3.08 (1.39)	2.52 (1.28)	0.02	3.2 (1.27)	3.21 (1.26)	3.17 (1.35)	0.74
Perceived risk of COVID-19 to family and friends	3.86 (1.15)	3.87 (1.14)	3.5 (1.26)	0.1	3.88 (1.12)	3.88 (1.11)	3.94 (1.19)	0.60
Perceived risk of COVID-19 to people in Ireland	3.98 (1.05)	3.99 (1.04)	3.65 (1.18)	0.1	3.97 (0.96)	3.96 (0.96)	4 (0.96)	0.68
Have enough information about COVID-19 symptoms	4.43 (0.95)	4.43 (0.95)	4.42 (0.83)	0.96	4.54 (0.85)	4.54 (0.86)	4.51 (0.8)	0.69
Have enough information about self-isolation	4.46 (0.94)	4.46 (0.94)	4.42 (0.94)	0.82	4.45 (0.93)	4.44 (0.95)	4.56 (0.83)	0.12
Have enough information about testing	4.33 (1.03)	4.34 (1.03)	4 (1.11)	0.09	4.23, (1.12)	4.22 (1.12)	4.27 (1.12)	0.59
Have enough information about contact tracing programme	3.95 (1.3)	3.96 (1.3)	3.88 (1.27)	0.73	3.88 (1.32)	3.88 (1.32)	3.88 (1.33)	0.97
I am concerned about passing coronavirus on to someone who might be at risk	4.57 (1.02)	4.57 (1.03)	4.79 (0.6)	0.05	4.57 (0.92)	4.56 (0.94)	4.63 (0.8)	0.35
My personal behaviour has an impact on how coronavirus spreads	4.61 (0.97)	4.6 (0.98)	4.73 (0.8)	0.38	4.70 (0.78)	4.7 (0.78)	4.71 (0.8)	0.90
Information from the Government about coronavirus can be trusted	4.28 (1.09)	4.28 (1.1)	4.39 (0.9)	0.49	4.27 (1.06)	4.29 (1.05)	4.16 (1.1)	0.20
Worry about COVID-19	2.71 (1.05)	2.7 (1.05)	2.97 (1.03)	0.13	2.68 (0.94)	2.68 (0.95)	2.66 (0.84)	0.78

a 5-point scale (1 = strongly disagree-5 = strongly agree).

## 4 Discussion

This study undertaken during the first year of the COVID-19 pandemic at a time of unprecedented change and before the development of vaccines provides three main findings. Firstly, very high levels of compliance with self-isolation for cases and with restriction of movement for contacts were reported across a range of different sociodemographic and risk characteristics. Secondly, there was a high level of knowledge among cases and contacts of the need to self-isolate once a positive result was received, with most reporting satisfaction with information received on symptoms, self-isolation, tracing, and testing and an attitude of high level of risk perception of COVID-19. Thirdly, despite this high compliance behaviour and positive knowledge and attitudes, some cases and contacts attended high transmission risk settings during their potential infectious period.

The level of compliance observed in our study compares favourably with other studies conducted during the COVID-19 pandemic [[22], [23], [24], [25], [26], [27], [28], [29]]. However, such comparisons are limited as these studies varied in many ways in terms of study design, population, and mode of administration. Our study is a secondary data analysis of a phone survey with other studies using a range of sources included online and phone surveys as well as analysis of administrative data. The timing of administration of our study limits the applicability of our findings to other time points in the pandemic. Most other studies also utilised a cross-sectional design with exception of CORSAIR, a large UK panel survey with multiple waves [31,32]. CORSAIR reported low self-reported rates of adherence to isolating, testing, and quarantining and low rates of recognition of the main symptoms of COVID [31,32].

Age was the only significant predictor of compliance behaviour in our study with the highest levels of compliance reported among the youngest and oldest age groups. This was likely influenced by national guidance including cocooning for older adults and the use of remote learning among third level institutions. A French study reported higher levels of non-adherent behaviours by older people [27], whereas CORSAIR reported higher levels of non-adherence by younger people [31,32]. Among Chinese children, adherence was higher in older children [29]. In our study, there was no evidence of a gender difference with similar levels of compliance reported by men and women. This contrasts with a French study which reported women were more likely to report non-adherence behaviours [27], whereas adherence was higher in women in Iran [23] and the UK [26,31,32]. In addition to age and gender, we assessed the impact of employment, education, household composition and chronic illness. While we found no association between these factors and compliance behaviour, we had limited power due to the overall high compliance levels. In Ireland, a voluntary approach to compliance was taken. General supports such as availability of home delivery for shopping and delivery of medicines existed but additional supports for those advised to self-isolate or restrict movements were not provided by the state. Some employers facilitated working from home with this varying across different sectors. While many employers such as the health service provided additional paid sick leave for those with COVID-19, this varied across employers and sectors. At the time the survey was undertaken there was no statutory sick pay, though a new scheme was introduced in 2023 to bring Ireland in line with other European countries that have mandatory paid sick leave for workers. Other studies reported varying impact of household, employment and educational characteristics. Among older Iranians, living alone did not affect compliance [23] whereas, a DRC study reported lower levels of adherence in those living with others or a partner [22]. In the UK, having a dependent child was associated with non-adherence [26,31,32]. A DRC study reported lower adherence in those with lower education levels and the unemployed [22]. In contrast, in France those with higher education levels were more likely to report non-adherence [27]. Lower socioeconomic grade, greater hardship, and working in a key sector were associated with non-adherence in the UK [26,31,32]. Very high compliance was reported in South Korea where the government introduced a national system of self-isolation and uses sanctions for enforcement [24]. The often conflicting findings across the existing literature emphasises the importance of understanding local contexts and cultures in developing public health interventions [38].

In our analysis, survey respondents reported high levels of satisfaction with information received on COVID-19. Most participants who took part in the survey confirmed receipt of information on self-isolation and being told for how long they needed to self-isolate. The high levels of knowledge and understanding may have contributed to the high levels of compliance observed in our study. According to a comparison of citizen’s adherence to COVID-19 mitigation recommendations in the United States, Kuwait and South Korea, a higher intensity of general health information sources for COVID-19 and knowledge on COVID-19 positively influenced self-reported adherence [28]. A study from France on attitudes in the population reported three-quarters of those surveyed agreeing with general quarantine [27]. In Brazil, 74.2% of respondents of an online survey (n = 45,161) reported adherence to physical contact restrictions, which possibly contributed to decreasing the spread of COVID-19 [25].

## 5 Strengths and limitations

This study recruited a large representative sample with data utilising the national database. However, while sufficiently large to assess overall compliance, there was a lack of power for subgroups. The survey instrument was developed using existing validated questionnaires [[31], [32], [33]] and administered by experienced trained interviewers. However, while self-report is recognised as a valid measure [39,40], compliance may have been overestimated due to social desirability bias [41]. As the field work coincided with elevated level of restrictions reducing the difference between what was permitted generally and for cases or contacts may have overestimated compliance. Heightened awareness among the public due to the restrictions may also have enhanced knowledge about COVID-19 symptoms and transmission.

Among cases referred for testing due to symptoms non-compliers reported a longer mean duration of time between symptom onset and test date than who were compliant. A limitation of the survey was that it did not ascertain whether this was due to a delay in seeking a test or in accessing the test, limiting any inferences. Cases and contacts resident in congregate settings such as nursing homes or direct provision centres were excluded from the sample for practical reasons limiting the generalisability of the findings and potentially excluding groups with lower compliance.

## 6 Conclusion

This study demonstrates consistently high levels of compliance behaviour, high levels of knowledge of disease transmission and satisfaction with information. However, a minority of respondents did not adhere to public health guidance, attending workplaces and other potential high transmission risk settings. Our findings emphasize the importance of ensuring that societal supports including access to paid sick leave, ability to work from home and support with practical needs are provided as part of outbreak management.

## Author statements

Ethical approval for this secondary data analysis was received from the social research ethics committee in University College Cork, Ireland. There are no funding or competing interests to declare.

## Declaration of competing interest

The authors declare that they have no known competing financial interests or personal relationships that could have appeared to influence the work reported in this paper.
