# Supplementary material for: Cross-sectional survey of compliance behaviour, knowledge and attitudes among cases and close contacts during COVID-19 pandemic
Source: Public Health Pract (Oxf). 2023 Feb 15;5:100370. doi: 10.1016/j.puhip.2023.100370 (PMC9930406; doi:10.1016/j.puhip.2023.100370)
Supplement: Multimedia component 1 [file mmc1.docx]

**Webmaterials**

1. **Questionnaire**

**(2) Webtables**

**Web Table 1(a)Characteristics of Cases Stratified by Compliance with Self-isolation**

|  |  |  | **Self-isolated** |  |  |
| --- | --- | --- | --- | --- | --- |
| **Characteristics** | | **Overall**  **N=1027**  **N (%)** | **Yes**  **N=992***  **N (%)** | **No**  **N=34***  **N (%)** | **p-value** |
| Gender | *Male* | 553 (53.8) | 532 (53.6) | 21 (61.8) | 0.45 |
|  | *Female* | 474 (46.2) | 460 (46.4) | 13 (38.2) |  |
| Age | *Mean (SD)* | 36.1 (17.4) | 36.1 (17.5) | 35.5 (14.4) | 0.78 |
| Region | *Dublin* | 221 (21.5) | 211 (21.3) | 9 (26.5) | 0.79 |
|  | *ROL* | 304 (29.6) | 294 (29.6) | 10 (29.4) |  |
|  | *Munster* | 263 (25.6) | 253 (25.5) | 10 (29.4) |  |
|  | *Connacht* | 115 (11.2) | 112 (11.3) | 3 (8.8) |  |
|  | *Ulster* | 124 (12.1) | 122 (13.2) | 2 (5.9) |  |
| Occupation | *Employed* | 607 (59.1) | 584 (58.9) | 23 (67.6) | 0.26 |
|  | *Unemployed* | 74 (7.2) | 70 (7.1) | 4 (11.8) |  |
|  | *Student/pupil* | 215 (20.9) | 209 (21.1) | 6 (17.6) |  |
|  | *Retired* | 59 (5.7) | 58 (5.8) | 0 (0) |  |
|  | *Home worker* | 55 (5.4) | 55 (5.5) | 0 (0) |  |
|  | *Other* | 17 (1.7) | 16 (1.6) | 1 (2.9) |  |
| Highest Education Level | *Primary* | 39 (3.8) | 39 (4.0) | 0 (0) | 0.81 |
|  | *Secondary* | 451 (44.3) | 435 (44.3) | 15 (44.1) |  |
|  | *Tertiary* | 528 (51.9) | 509 (51.8) | 19 (55.9) |  |
| Household size | *Median (IQR)* | 4 (3, 5) | 4 (3,5) | 3 (3, 4.75) | 0.9 |
| Child in household | *Child present* | 424 (43.7) | 409 (43.6) | 15 (46.9) | 0.86 |
|  | *None* | 546 (56.3) | 528 (56.4) | 17 (53.1) |  |
| Long-term health condition | *Present* | 203 (19.8) | 195 (19.7) | 8 (23.5) | 0.74 |
|  | *None* | 822 (80.2) | 795 (80.3) | 26 (76.5) |  |
| Native English speaker | *Yes* | 970 (94.4) | 938 (94.6) | 31 (91.2) | 0.43 |
|  | *No* | 57 (5.6) | 54 (5.4) | 3 (8.8) |  |
| *one case did not provide information about self-isolation status after testing positive | | | | | |

**Webtable 1 (b) Characteristics of Close Contacts referred for testing due to close contact stratified by compliance with movement restriction**

|  |  | **Contacts N=1078** | **Restricted Movements N=933*** | **Did Not Restrict Movements N=140*** | **p-value** |
| --- | --- | --- | --- | --- | --- |
| Gender | *Male* | 501 (46.6) | 429 (46.1) | 69 (49.3) | 0.54 |
|  | *Female* | 575 (53.4) | 502 (53.9) | 71 (50.7) |  |
|  |  |  |  |  |  |
| Age | Mean (SD) | 36.33 (19.22) | 36.24 (19.43) | 36.99 (17.85) | 0.65 |
| Region | *Dublin* | 212 (19.7) | 182 (19.5) | 29 (20.7) | 0.98 |
|  | *ROL* | 247 (22.9) | 217 (23.3) | 30 (21.4) |  |
|  | *Munster* | 371 (34.4) | 320 (34.3) | 48 (34.3) |  |
|  | *Connacht* | 148 (13.7) | 127 (13.6) | 20 (14.3) |  |
|  | *Ulster* | 100 (9.3) | 87 (93) | 13 (9.3) |  |
| Occupation | *Employed* | 611 (56.7) | 529 (56.7) | 80 (57.1) | 0.15 |
|  | *Unemployed* | 72 (6.7) | 57 (6.1) | 15 (10.7) |  |
|  | *A student or pupil* | 216 (20) | 191 (20.5) | 23 (16.4) |  |
|  | *Retired* | 81 (7.5) | 73 (7.8) | 7 (5.0) |  |
|  | *Homemaker* | 66 (6.1) | 58 (6.2) | 8 (5.7) |  |
|  | *Other* | 32 (3.0) | 25 (2.7) | 6 (5.0) |  |
| Highest Education level | *Primary* | 75 (7) | 63 (6.8) | 12 (8.6) | 0.74 |
|  | *Secondary* | 473 (44.4) | 414 (45) | 57 (41) |  |
|  | *Tertiary* | 517 (48.5) | 444 (48.2) | 70 (50.4) |  |
| Household size | *Median (IQR)* | 4 (3, 5) | 4 (3, 5) | 3 (2, 4) | 0.12 |
| Child in household | *Child present* | 444 (43.6) | 395 (44.8) | 47 (35.6) | 0.06 |
|  | *None* | 574 (56.4) | 486 (55.2) | 85 (64.4) |  |
| Long-term condition | *Present* | 203 (18.9) | 179 (19.2) | 23 (16.7) | 0.54 |
|  | *None* | 870 (81.1) | 751 (80.8) | 115 (83.3) |  |
| Native English speaker | *Yes* | 1032 (95.7) | 893 (95.7) | 135 (96.4) | 0.82 |
|  | *No* | 46 (4.3) | 40 (4.3) | 5 (3.6) |  |
| *five contacts did not provide information about restriction of movement | | | | | |

**Webtable 2 Multivariable logistic regression model of non-compliance**

| **Characteristics** | **Adjusted Odds Ratio*** | **95% CI** | **p-value** |
| --- | --- | --- | --- |
| **Age** (vs. <20) 20-29 30-39  40-49  50-59  60-69  70+ | 1.89  2.68  2.27  1.89  5.2  2.29 | 1.03-3.43  1.38-5.19  1.15-4.45  0.95-3.75  2.53-10.75  0.75- 6.94 | 0.04  0.003  0.017  0.070  0.001  0.144 |
| **Gender** (vs. male) | 1.04 | 0.74-1.45 | 0.83 |
| **Education** (vs. primary) Secondary  Tertiary | 1.50  1.42 | 0.58-3.89  0.53-3.79 | 0.40  0.48 |
| **Employed** (working vs. not) | 1.18 | 0.80-1.74 | 0.41 |
| **Child in Household** | 1.00 | 0.69-1.46 | 0.99 |
| **Long-term Condition** | 0.96 | 0.63-1.47 | 0.87 |
| **Non-native English speaker** | 0.89 | 0.45-1.76 | 0.75 |

* OR adjusted for all other variables in the table
